# Understanding Drug and Alcohol Staff Perspectives on the Barriers and Facilitators to Drug Checking: A Qualitative Study

**DOI:** 10.1111/dar.14073

**Published:** 2025-05-04

**Authors:** Nina Pocuca, Brodie C. Dakin, Cheneal Puljević, Cameron Francis, Daniel Stjepanović, Anthony Barnett, Leanne Hides

**Affiliations:** ^1^ National Centre for Youth Substance Use Research, School of Psychology, Faculty of Health, Medicine and Behavioural Sciences The University of Queensland Brisbane Australia; ^2^ Centre of Research Excellence on Achieving the Tobacco Endgame School of Public Health, Faculty of Health, Medicine and Behavioural Sciences, The University of Queensland Brisbane Australia; ^3^ The Loop Australia Brisbane Australia; ^4^ Lives Lived Well Brisbane Australia

**Keywords:** alcohol and other drug staff perspectives, drug checking, harm reduction, people who use drugs

## Abstract

**Introduction:**

Drug checking (i.e., whereby members of the public submit a drug sample for pharmacological analysis of the drug content) is an evidence‐based harm reduction tool. Despite this, the uptake of drug checking services by people who use drugs (PWUD) is often limited across different jurisdictions and types of services, highlighting the need for research examining barriers to drug checking uptake from the perspective of key stakeholders. This qualitative pre‐implementation study explored the perspectives of staff employed by alcohol and other drug (AOD) organisations on drug checking, including barriers and facilitators to uptake.

**Methods:**

Interviews were conducted with 23 AOD harm reduction and AOD treatment staff (14 female; mean age = 38.8 years, SD = 8.2). Qualitative data were analysed using iterative categorisation.

**Results:**

Five themes were extracted from the data: (i) PWUD infrequently are most likely to access drug checking; (ii) Confidentiality and anonymity concerns are barriers to drug checking; (iii) Ease of use is integral to drug checking uptake; (iv) Safe, non‐judgemental environments that include peer workers are critical; and (v) People who sell drugs will likely use drug checking.

**Discussion and Conclusions:**

The following factors were identified as paramount to the uptake of drug checking services among PWUD: (i) confidentiality; (ii) agreements or memoranda of understanding that protect service clients from over‐policing and criminalisation; (iii) mobile and fixed‐site services that are accessible to PWUD; and (iv) a non‐judgemental and safe environment that includes both health professionals and peer workers with lived experience.

**Summary**

- Four factors were identified as key to the uptake of drug checking services: confidentiality; agreements to protect clients from criminalisation; accessible mobile and fixed‐site services; and a non‐judgemental and safe environment including both health and peer workers.

## 1 Introduction

The consumption of drugs produced in an unregulated market significantly increases human health and mortality risks already posed by regulated substances [1, 2]. Beyond the inherent risks associated with substance use, risks of using drugs produced in unregulated markets include consuming larger‐than‐intended drug doses or unwittingly consuming other hazardous substances contained in the drug such as fentanyl, xylazine, *N*‐ethylpentylone, or nitazenes, which have been linked to hospitalisations and fatalities [3, 4, 5, 6]. Given the harms associated with drug use and in the context of drug prohibition, there has been a global push to introduce drug checking as a harm reduction tool [7, 8, 9, 10].

Drug checking is the process whereby members of the public submit a drug sample (usually a scraping or one‐tenth of a gram from a pill, powder, crystal or blotter) for pharmacological analysis of the drug content (and purity in some instances), with the results returned to the service user via a tailored intervention that aims to reduce drug‐related harms [11]. Drug checking services operate in at least 26 countries across Europe, the Americas and Australasia [12]. Importantly, studies investigating drug checking at music festivals estimate that between 12% and 27% of submitted drug samples did not contain the drug expected by the service user, and drug checking at music festivals, fixed‐site and community‐based services have detected the presence of potentially harmful substances in drug samples (e.g., fentanyl, phenacetin, *N*‐ethylpentylone and nitazenes) [5, 6, 13, 14, 15, 16, 17, 18, 19]. In turn, the identification of unexpected and potentially harmful substances via drug checking has been associated with increased harm reduction behaviours, including drug disposal or consumption of a smaller dose of the drug [13, 14, 15, 17, 18, 19].

Despite the clear benefits of drug checking services and the fact that some drug checking services are well‐attended (e.g., the Substance Drug Checking service in Canada received > 900 samples each month from May to July 2024 [20] and Drugs Information and Monitoring System in the Netherlands received > 19,000 samples in 2023 [21]), overall uptake of drug checking services among people who use drugs (PWUD) is still limited. For instance, only 1%–3% of PWUD accessed available drug checking services at English music festivals or utilised available fentanyl test strips at a supervised drug consumption facility in Vancouver [15, 16, 22]. These low uptake rates often seen for drug checking services are surprising considering previous research found between 43% and 94% of PWUD had the intention of using a drug checking service, if available [23, 24, 25], particularly if certain service characteristics were met (e.g., police supported the service by keeping their distance and not disrupting operation; ≤ 20 min wait time) [25, 26, 27]. These findings highlight the need for research examining barriers to drug checking uptake.

Human‐centred co‐design approaches that engage persons with lived experience and key stakeholders in: (i) identifying barriers to the implementation and uptake of health interventions; and (ii) co‐creation of interventions that address these barriers, have been associated with increased user acceptance and engagement [28, 29, 30]. While engaging PWUD in the co‐design of a drug checking service is critical, it is also important to understand the perspectives of people working in the alcohol and other drug (AOD) sector, given the critical role service providers play in the successful implementation and uptake of health interventions [31]. For example, key implementation science frameworks such as the Consolidated Framework for Implementation Research outline the importance of staff perceptions of intervention characteristics as integral to the successful implementation of a health intervention [32]. Namely, the Consolidated Framework for Implementation Research posits that factors including whether staff believe the intervention was developed internally (e.g., with consultation from staff in the AOD sector), beliefs about the quality, complexity and design of the intervention and whether it will achieve the intended outcomes are vital to the successful implementation of a health intervention. These factors underscore the value of eliciting AOD staff perspectives on drug checking to ultimately facilitate successful implementation.

Only two studies—both based in the US—have examined the barriers and facilitators to drug checking from the perspective of AOD staff. One study of staff at a needle and syringe exchange programme identified the unclear nature regarding the legality of drug checking services and the complexity of administering drug checking and interpreting results as significant barriers to implementation [33]. Similarly, a qualitative study examining perspectives on implementing fentanyl drug checking among 32 AOD workers found implementation barriers including issues with legality and the underlying need for trust and rapport between the drug checking service providers and PWUD [34]. While these two studies provide important insights into the barriers and facilitators for drug checking, only one study [34] was conducted prior to implementing drug checking. Implementation science frameworks champion the importance of pre‐implementation studies as pivotal to increasing understanding of potential barriers and facilitators to inform intervention and implementation design and ultimately, increase the likelihood of successful intervention uptake [32, 35]. Thus, further pre‐implementation research is needed to understand AOD staff perspectives on the barriers and facilitators to drug checking for different populations of PWUD, including those who use drugs infrequently (e.g., at music festivals) and those who are dependent on drugs. It is also particularly important to explore perspectives of Australian AOD staff on drug checking in light of newly established drug checking services in three states as of January 2025 [36, 37, 38, 39]; however, such research is currently lacking. Thus, this qualitative pre‐implementation study aimed to explore Australian AOD staff perspectives on drug checking, including barriers and facilitators to the uptake of drug checking among PWUD.

## 2 Method

### 2.1 Setting

This study was conducted between February and August 2023. At this time, Australia had one fixed‐site service and two trials of drug checking services conducted at music festivals (all occurring in Canberra, Australia). While drug checking services were approved for operation in the state of Queensland in February 2023 [36], this study occurred before the opening of the state's first fixed‐site service in April 2024 [39]. At the time of this study, no other states had passed legislation approving either fixed‐site or music festival‐based drug checking services in Australia.

Participants comprised a convenience sample of staff recruited from three separate not‐for‐profit AOD organisations in Australia: (i) Lives Lived Well (LLW); (ii) Queensland Injectors Health Network (QuIHN); and (iii) The Loop Australia. LLW is a large AOD treatment provider in Australia, while QuIHN is a peer‐ and expert‐led organisation providing harm reduction and health services to PWUD, including needle and syringe exchange programmes. The Loop Australia aims to develop and implement the sustainable provision of drug checking services in Australia and is comprised of volunteer chemists, medical doctors, social workers and other allied health professionals.

### 2.2 Participants

Participants were invited to participate in the present study if they worked in a client‐facing role or had experience delivering drug checking services. The final sample comprised 23 staff (LLW *n* = 10, QuiHN *n* = 3, The Loop Australia *n* = 10; see Table 1 for descriptive statistics). To maintain participant confidentiality and given their joint focus on harm minimisation, QuIHN and The Loop Australia staff are referred to herein as AOD harm reduction staff, whereas LLW staff are referred to herein as AOD treatment staff.

**TABLE 1:** Participant characteristics.

Variable	*M* (SD)/% (*n*)
Age, *M* (SD)	38.8 (8.20)
Female, % (*n*)	61% (14)
Highest degree	
Certificate or diploma	9% (2)
Bachelors	52% (12)
Postgraduate degree (i.e., Masters or post‐graduate diploma)	39% (9)
Professional experience with drug checking	13% (3)

Abbreviations: M, mean; SD, standard deviation.

### 2.3 Procedure

This study followed the consolidated criteria for reporting qualitative studies (COREQ) checklist (see Tables S1, Supporting Information) [40]. Qualitative methods were adopted given their fluid and rigorous nature allows for the provision of detailed information on the implementation context and environment, aiding in the identification of potential barriers and facilitators to implementation and laying the groundwork for successful implementation [41]. Participants were recruited via organisation‐wide emails. Participants first completed a 15‐min online survey and were then invited to participate in a semi‐structured qualitative interview. The present study reports on the results of the qualitative interviews. All but one interview (conducted in‐person) was conducted online via Zoom, with only the interviewer and participant present. Interviews ranged from 21 to 51 min in duration (*M* = 34 min; SD = 9 min). Interviews were audio‐recorded with participant consent to allow for transcription and were conducted by one male and one female member of the research team (B.C.D. and M.C.) who were employed as research assistants. Both interviewers were completing their postgraduate clinical psychology degrees, had experience working with clients, and were trained in qualitative interviewing. Further information about the research team is provided in the Supporting Information. Participants were reimbursed $20 for their time.

Participants were asked about: (i) their work or volunteer experience in the AOD sector; (ii) their professional experience with drug checking; (iii) the likelihood that drug checking will be accessed by different cohorts of PWUD (e.g., PWUD infrequently, compared to people who are dependent on drugs); (iv) perceived barriers and facilitators to PWUD accessing drug checking; and (v) how they feel about drug checking being co‐located at different AOD services (see Supporting Information for the interview guide). Interviews were conducted until data saturation was obtained, as determined by consensus between the interviewers and research team [42]. Audio recordings were transcribed and de‐identified to preserve confidentiality. Study procedures were approved by The University of Queensland Human Ethics Research Committee (2022/HE002006) and research was performed in compliance with relevant laws and institutional guidelines.

### 2.4 Data Analysis

Qualitative data were analysed using iterative categorisation [43, 44] and conducted in NVivo (Release 1.7). First, the primary coder (B.C.D.) coded all interviews using the semi‐structured interview protocol as a guide (i.e., deductive approach to qualitative analyses). Analyses and interpretation of the data were not based on an existing theoretical framework but were instead based on existing drug checking literature and authors' experience within this field. Deductive analyses were supplemented by an inductive approach whereby the coder also allowed the creation of ideas that came‐up organically, rather than in response to specific interview questions. N.P. then used a qualitative codebook developed by B.C.D. (including all the codes for analyses and associated descriptions) to independently code five interviews (22%). Intercoder reliability was examined using Cohen's Kappa to ensure code clarity and decrease the likelihood of a single author influencing analyses [45]. B.C.D. then derived preliminary themes from the data which were iteratively reviewed and revised by N.P. and B.C.D. to derive the final themes.

## 3 Results

Five themes were identified. These themes are discussed below and appear in Table S2 with de‐identified example quotes from participants.

### 3.1 Theme 1: People Who Use Drugs Infrequently Are Most Likely to Access Drug Checking

Both AOD harm minimisation and treatment staff noted differences in the likelihood to access a drug checking service between PWUD infrequently (e.g., at a music festival) and people who are dependent on drugs. All staff emphasised that drug checking would be popular among people using drugs infrequently, given the prevalence of the harm minimisation discourse and acceptance of drug checking among groups of PWUD at festivals internationally. It was highlighted that people who use substances infrequently may also have a heightened perception of their vulnerability to harm from drug use given: (i) potential use of different sources to procure their drugs, which may mean they have less confidence surrounding the content or quality of the drugs; (ii) their lower physiological tolerance for drugs; and (iii) generally more limited knowledge about how to use drugs in a way to reduce the risk of harm. Finally, it was noted that practically, this group would have the financial resources to use drug checking services:

I think in terms of festivals, for people who use recreationally and who understand about harm minimisation, who are not necessarily dependent on substances … they're the category who I would imagine would be the most likely to use [drug checking]. 50 years, AOD treatment staff

Many harm minimisation staff emphasised the unique needs and barriers to accessing a drug checking service faced by people who are dependent on drugs. First, it was noted that people who are dependent on drugs generally have fewer financial resources and so may be more deterred by having to relinquish a portion of their drug for testing. Second, staff mentioned psychological barriers to accessing a drug checking service, wherein people who are dependent on drugs may perceive less need to use drug checking, because they are: (i) less risk averse; (ii) more likely to have developed tolerance and therefore are less vulnerable to drug purity; and (iii) more familiar with the drug they are using. Staff also noted that people who are dependent on drugs may have a higher sense of urgency to use drugs to avoid withdrawal symptoms and therefore be less willing to delay use and test their drugs beforehand:

Financially, someone who is homeless, on benefits, and dependently injecting heroin, would be a good example. I would say, it would be less likely someone like that would pay for [drug checking] just from a resource point of view. 45 years, AOD harm reduction staff

Nevertheless, staff postulated that some PWUDs dependently may access drug checking if it were located within services with which they have existing relationships (see Theme 3 for further details).

### 3.2 Theme 2: Confidentiality and Anonymity Concerns Are Barriers to Drug Checking

Almost all staff (20 of 23) cited confidentiality concerns as a significant barrier to people accessing drug checking. Primarily, staff posited a fear of being targeted by the police as someone using or carrying illicit drugs and the anticipated resulting legal consequences as the primary confidentiality concern. Having a poor prior history with police was cited as underscoring the fear of police presence and intervention, particularly among people with a history of frequent or dependent drug use. Staff also cited the fear of being seen by others (e.g., family, friends, work colleagues or just society at large) entering or exiting a drug checking service may also be a deterrent for accessing drug checking, particularly among people who use substances less frequently:

I do think you still have a struggle of the clients that really just don't believe that police aren't involved, you know, that the minute they hand those drugs in, a police officer isn't gonna snatch them out the door or catch them walking in. 26 years, AOD treatment staff

Participants also discussed how to allay confidentiality concerns. Three AOD staff highlighted the importance of limiting the questions clients accessing drug checking are asked, or allowing them to complete drug checking anonymously. Another highlighted the importance of ensuring clients were not crossing paths in the waiting area of the drug checking service by having private waiting and testing cubicles where clients enter via one door and leave via a separate door. Finally, another participant mentioned making the service discreet or possibly locating it within a broader health service:

Fixed‐sites, are people going to feel comfortable attending? Are they going to feel like people are watching them going in and out? I don't know … Is the fixed‐site joined to a health service or something else? So [drug checking] is not your sole purpose for going in is everybody knows that where people go to check drugs. 48 years, AOD harm reduction staff

### 3.3 Theme 3: Ease of Use Is Integral to Drug Checking Uptake

Staff highlighted the importance of location and wait time as key accessibility factors implicated in the likely uptake and success of drug checking. Appropriate and convenient locations included mobile sites at or near festivals, as well as fixed sites co‐located within existing health services. It was also highlighted as important for fixed‐site drug checking services to be easily accessible by public transport or in a location frequented by PWUD:

If it's not accessible, and if it's not something easy, a lot of people are not going to go out of their way [to use it]. 52 years, AOD harm reduction staff

It would have to be instant results I think. I don't think people are gonna want to wait. They want to take the drugs. 36 years, AOD treatment staff

Co‐location of drug checking services within a site providing a needle and syringe programme was mentioned by both harm reduction and treatment staff as suitable. This co‐location was seen as particularly important to facilitate access for people who are dependent on drugs, given convenience and increased anonymity and confidentiality, as it would be ambiguous whether an individual was accessing drug checking or another health service (important facilitator identified in theme 2). Finally, another benefit of co‐locating drug checking within an AOD treatment or health service is that it may also make it easier for people to access AOD treatment. There was some concern, however, at the idea of co‐locating drug checking within a drug and alcohol treatment service; as such a service may also be catering to people seeking to abstain from substances. Finally, it was noted that while co‐locating at needle and syringe programmes may facilitate engagement for people who are dependent on drugs, it may also deter PWUD infrequently from accessing drug checking due to the stigma associated with injecting drug use.

### 3.4 Theme 4: Safe, Non‐Judgemental Environments That Include Peer Workers Are Critical

Staff identified that fostering a safe and non‐judgemental environment is paramount to drug checking uptake. Staff mentioned that having peer workers with lived experience employed at the drug checking service, working alongside or as health professionals would lead to a reduction in perceived stigma and judgement, increased trust, and ultimately make people more comfortable accessing the service. While staff recognised the benefits of including health professionals in the service, several mentioned that having to interact with a health professional (rather than a peer) may be a barrier for many PWUD, due to past negative experiences with health systems and fear that they will be judged or stigmatised for their substance use:

… One thing that we hear all the time is how powerful and how needed the peer work is. And it's also like modelling that harm minimization, if it's someone who's still using drugs, but also like recovery, it can be also giving hope, you know, around recovery, just seeing someone who has been there been in addiction and then out of it can be really motivating. 38 years, AOD harm reduction staff

### 3.5 Theme 5: People Who Sell Drugs Will Likely Use Drug Checking

Though there were no specific questions about people who sell drugs in the interview protocol, several staff spontaneously mentioned the high likelihood and implications of people who sell drugs accessing drug checking. Several harm reduction staff mentioned that people who sell drugs would be better informed of what they are selling if they tested their drugs, which would allow them to make informed choices about what they choose to sell, or provide them with information they can pass on to their customers about the contents of the drugs they are selling, facilitating customers' ability to make informed decisions about what they purchase and consume. Some AOD staff thought that this would serve to improve the quality of the illicit drug market:

I think if you're getting people who sell drugs on board with drug checking service, like ultimately, you know, you're helping them be more informed about their own substances that they're selling. And potentially, that would filter down to the consumer … 32 years, AOD harm reduction staff

In contrast to the potential positive outcomes, some AOD treatment staff hypothesised that people who sell drugs who use drug checking would not be motivated by the best interests of their customers, but rather be motivated to maximise profits by cutting high‐purity drugs with other substances or selling them at a greater price:

… low level, dealers, traffickers that may use the services to assess the purity of their substances or what they have for the benefit of understanding what they're selling …Not for informing their customers, but to go ‘Well I'm on to a great batch of coke here’ …and drive their pricing. 34 years, AOD treatment staff

## 4 Discussion

This qualitative, pre‐implementation study examined AOD staff perspectives on drug checking, including barriers and facilitators to the uptake of drug checking among PWUD. Five themes were identified: (i) PWUD infrequently are most likely to access drug checking; (ii) confidentiality and anonymity concerns are barriers to drug checking; (iii) ease of use is integral to drug checking uptake; (iv) safe, non‐judgemental environments that include peer workers are critical; and (v) people who sell drugs will likely use drug checking.

Both harm reduction and AOD treatment staff hypothesised that PWUD frequently or people who are dependent on drugs would be less likely to use drug checking than PWUD infrequently (e.g., at festivals or parties), potentially due to a lower perceived risk of drug use and a greater urgency to use drugs to alleviate or avoid withdrawal symptoms. These ideas have been echoed in other studies by PWUD [26, 46], though these findings may be contextual. That is, in unregulated markets where adulterants such as fentanyl are very common, PWUD have shown interest (albeit at varying levels of enthusiasm) in and repeat engagement with drug checking services [47, 48]. Ultimately, this study and previous research posited differences between PWUD frequently versus infrequently in their likelihood to access drug checking; however, studies have found similar rates of uptake of available drug checking services (between 1% and 3%) offered at festivals and supervised drug consumption facilities (whose clients frequently inject drugs) [15, 16, 22]. In line with some studies examining potential service user perspectives on drug checking [27] and adding further to the AOD staff literature surrounding barriers to drug checking [33, 34], AOD staff in the present study identified potential client financial constraints associated with surrendering a drug sample as a barrier to accessing a drug checking service for marginalised groups of PWUD (e.g., people who are dependent on drugs or are homeless).

Confidentiality and anonymity concerns were repeatedly cited by AOD staff as the greatest barrier to drug checking. This finding echoes sentiments reported previously by both AOD staff working within [33] and PWUD accessing [25, 27] a drug checking service. While confidentiality concerns identified in the present study were predominantly mentioned in the context of police intervention, they also extended to a fear of being identified by family, friends, or work colleagues while using a drug checking service, given the stigma surrounding drug use. Thus, these findings collectively point to not only the need for confidentiality and legal protection for PWUD checking services, but highlight the need for discreet drug checking services such as mail‐in services or inconspicuous drop‐off points, as suggested in a review of community‐based drug checking services [49].

In line with the review of community‐based drug checking services [49], the present study identified that a user‐friendly drug checking service was integral to uptake, with a recognised need for efficient and easily accessible services. As a result, participants recognised a need for mobile (e.g., at a music festival or nightclub entertainment precinct) and easily accessible (e.g., via public transport) fixed‐site drug checking services. Particularly, co‐locating a fixed‐site drug checking service within existing harm reduction services (e.g., a needle and syringe exchange programme) was seen as beneficial, given they have established relationships with PWUD and allow for easy linking of new service clients with other harm reduction resources.

The present study also identified that a service comprised of both peer workers and health service professionals is paramount to a successful and non‐judgemental drug checking service that fosters trust with PWUD. These findings are in line with existing research which found the integration of peers into AOD services was associated with both client and organisational benefits, including the development of stronger professional relationships between clients and non‐peer health professionals [49].

AOD treatment and harm reduction staff discussed the implications of people who sell drugs accessing drug checking services. Harm reduction staff highlighted potential harm minimisation implications (e.g., to identify adulterated drugs and influence selling behaviours), while AOD treatment staff generally viewed that accessing a service for people selling drugs would do little to influence their behaviours. In other work exploring the perceptions of harm reduction staff, a qualitative study of 14 people who used a drug checking service and who sold drugs in the past month found the primary motivation for this group to test their drugs was to inform their customers about the contents of the drugs and to dilute the drug to make it safer for their clients to consume (if fentanyl was found to be present) [50]. These findings are supported by other studies, which reiterate the importance of PWUD maintaining long‐term relationships with reputable and trustworthy people to sell them drugs to reduce drug‐related harm [46, 51]. However, beyond a consumer/seller binary, and representing complex drug markets with different actors, people accessing drug checking services may be consumers, sellers, or both [52]. In addition to harm reduction benefits for individuals, other work has identified wider benefits of engaging people who sell drugs in drug checking services, including assisting in identifying particularly large quantities of adulterated product within a market, and the potential for sellers to engage in harm reduction practices to benefit public health (e.g., for people who sell drugs to access and distribute naloxone as for form of overdose prevention) [53]. Providing training and education to workers from different backgrounds who deliver and support drug checking services is important to ensure that individual and public health benefits are realised for people who access drug checking who play different (and multiple) roles in drug markets including consumption, selling and both, at varying times. It is acknowledged, however, that this is a challenging conversation, particularly given that it is likely that people who sell drugs accessing drug checking remains a difficult concept politically and for the general public.

### 4.1 Practical Implications

The findings of the present study suggest the combination of several critical factors for the successful implementation and uptake of drug checking services. First, services require design features geared towards promoting confidential and anonymous service use. Locating checking services within existing health care or harm reduction services, and providing take‐home drug checking where this is possible, have been noted as means to increase anonymity that are acceptable to PWUD [54, 55, 56]. Second, whether achieved via legal means and/or informal memoranda of understanding, it is important that there are co‐operative agreements between health policymakers, police and drug checking service operators that aim to protect clients from over‐policing, criminalisation and legal repercussions. Third, both mobile and fixed‐site drug checking services that are optimally positioned to be accessible to PWUD should be available (e.g., at music festivals and within established harm reduction services). Fourth, services need to include both qualified health and scientific professionals (e.g., chemists and health workers) and peer workers with lived experience to foster a non‐judgemental and safe environment for PWUD. Lastly, there is a need for education of both AOD staff and people who sell drugs regarding the potential role of drug checking in the creation of a safer drug supply. A qualitative ethnographic study of people who sell drugs in Vancouver, Canada, found participants translated the results of drug checking into more interpretable language (e.g., percentage of fentanyl detected to “strong” or “weak”) when selling to PWUD [50]. Given this behaviour, it would be helpful to educate people who sell drugs on how to interpret findings from drug checking to ensure that the information they convey to their purchasers is sufficiently accurate to enable harm reduction.

Ultimately, the AOD staff interviewed in the present study perceived that only a holistic approach to drug checking that ensures confidentiality, police and legal protections, accessibility and safe, peer‐integrated environments that focus on decreasing stigma and promoting safer drug supply would result in a successful drug checking service.

### 4.2 Strengths and Limitations

Strengths include the employment of the pre‐implementation qualitative interview method, which allowed a thorough investigation of the barriers and facilitators to the successful implementation and uptake of a drug checking service, from the perspective of AOD staff. Staff perspectives are vital as they exert a strong influence on the successful implementation and uptake of health service interventions [31]. Nonetheless, present findings should be considered in combination with perspectives from PWUD, to allow for a comprehensive understanding of the factors vital to the successful implementation and uptake of drug checking. Limitations include the limited sample size (*N* = 23); however, small sample sizes are usual in qualitative research [46, 50, 57]. Further, the results of this study are strengthened through the inclusion of a diverse range of AOD staff, including those with experience working in harm reduction settings (e.g., needle and syringe exchange programmes; *n* = 13) and AOD treatment (*n* = 10). A limitation of pre‐implementation research is the lack of experience with the health intervention among participants. To combat this, we provided explanations surrounding aspects of drug checking and descriptions of how the services have operated elsewhere (e.g., in pilot festival trials and at CanTEST), so that participants could envisage what the process may look like and consider potential barriers and facilitators. Finally, a limitation of drug checking itself may be a reluctance by PWUD to give up a sample of their drugs for testing given the already small drug amounts individuals are generally purchasing. One promising way around this may be through substance residue analysis, an alternative to drug checking piloted in Finland [58].

## 5 Conclusions

Drug checking is an evidence‐based harm reduction strategy [13, 14, 15, 17, 18, 19]. The present study identified several potential barriers and facilitators to the successful implementation and uptake of drug checking services by PWUD, including confidentiality and anonymity concerns (particularly in relation to police), ease of use, and the need for the integration of peer workers. In turn, present findings suggest mobile and fixed‐site drug checking services with design features that ensure confidentiality, limit police presence and protect service clients from legal repercussions, as well as include both peer workers and health professionals, are integral to the successful implementation of drug checking within the Australian context. Ultimately, findings from the present study should be integrated with findings from interviews conducted with PWUD to develop a drug checking service that has the maximum likelihood of successful implementation.

## Author Contributions

Each author certifies that their contribution to this work meets the standards of the International Committee of Medical Journal Editors. N.P. contributed to conceptualisation, formal analysis, methodology, project administration, supervision, validation, writing – original draft and review and editing; B.C.D. contributed to formal analysis, writing – original draft and review and editing; C.P. contributed to conceptualisation, methodology, writing – review and editing; C.F. contributed to conceptualisation, methodology, writing – review and editing; D.S. contributed to writing – review and editing; A.B. contributed to writing – review and editing; L.H. contributed to conceptualisation, funding acquisition, methodology, supervision, writing – review and editing.

## Conflicts of Interest

The authors declare no conflicts of interest.

## Supporting information


Data S1.
