# Supplementary material for: Understanding Drug and Alcohol Staff Perspectives on the Barriers and Facilitators to Drug Checking: A Qualitative Study
Source: Drug Alcohol Rev. 2025 May 4;44(5):1330–8. doi: 10.1111/dar.14073 (PMC12228017; doi:10.1111/dar.14073)
Supplement: Supplementary file 1 — Data S1. [file DAR-44-1330-s001.docx]

**Supporting Information**

# Table S1. Consolidated criteria for reporting qualitative studies (COREQ): 32-item checklist

| **Item number** | **Topic** | **Guide questions/description** | **Reported page/s** |
| --- | --- | --- | --- |
| **Domain 1: Research team and reflexivity** | | |  |
| *Personal Characteristics* | | |  |
| 1 | Interviewer/facilitator | Which author/s conducted the interview or focus group?  **BCD and MC conducted the interviews.** | 9-10 |
| 2 | Credentials | What were the researcher’s credentials? E.g. PhD, MD  **NP – PhD**  **BCD – MPsych (Clin), PhD**  **CP – PhD**  **CF – Bachelor of Social Work, Master of Health Studies (Addiction Studies)**  **DS – PhD**  **LH – PhD (Clin Psych)** | 9-10 |
| 3 | Occupation | What was their occupation at the time of the study?  **NP – Research Fellow**  **BCD – Research Assistant**  **CP – Senior Research Fellow**  **CF – CEO of The Loop Australia**  **DS – Senior Research Fellow**  **LH – Professor** | 9-10 |
| 4 | Gender | Was the researcher male or female?  **One interviewer was male (BCD), the other was female (MC)** | 9-10 |
| 5 | Experience and training | What experience or training did the researcher have?  **Both interviewers had a bachelor (honours) degree in psychology (BCD also had a PhD in Psychology), were completing their postgraduate masters of clinical psychology degree, had experience working with clients, and were trained in qualitative interviewing.** | 9-10 |
| *Relationship with participants* | | |  |
| 6 | Relationship established | Was a relationship established prior to study commencement?  **The interviewers and those involved in analysing the interview data did not have established relationships with any of the study participants** | 9-10 |
| 7 | Participant knowledge of the interviewer | What did the participants know about the researcher? e.g. personal goals, reasons for doing the research  **The consent form outlined that the study aimed to better understand the potential barriers and facilitators to accessing drug checking services, perceptions of drug checking services, and which features of a drug checking service were most appealing or useful for potential service clients.** | 7-8 |
| 8 | Interviewer characteristics | What characteristics were reported about the interviewer/ facilitator? e.g. Bias, assumptions, reasons and interests in the research topic  **The consent form outlined that the study aimed to better understand the potential barriers and facilitators to accessing drug checking services, perceptions of drug checking services, and which features of** **a drug checking service were most appealing or useful for potential service clients.** | 7-8 |
| **Domain 2: study design** | | |  |
| *Theoretical framework* | | |  |
| 9 | Methodological orientation and theory | What methodological orientation was stated to underpin the study? e.g. grounded theory, discourse analysis, ethnography, phenomenology, content analysis  **The present study used iterative categorisation.** | 9-10 |
| *Participant selection* | | |  |
| 10 | Sampling | How were participants selected? e.g. purposive, convenience, consecutive, snowball  **Convenience sample** | 7-8 |
| 11 | Method of approach | How were participants approached? e.g. face-to-face, telephone, mail, email  **Email.** | 7-8 |
| 12 | Sample size | How many participants were in the study?  **N=23** | 7 |
| 13 | Non-participation | How many people refused to participate or dropped out? Reasons?  **No participants dropped out of the study. Participants self-selected to participate in the research.** | n/a |
| *Setting* | | |  |
| 14 | Setting of data collection | Where was the data collected? e.g. home, clinic, workplace  **Via Zoom (+1 face-to-face interview)** | 7-8 |
| 15 | Presence of non-participants | Was anyone else present besides the participants and researchers?  **No.** | 7-8 |
| 16 | Description of sample | What are the important characteristics of the sample? e.g. demographic data, date  **The final sample comprised N=23 staff (LLW n=10, QuiHN n=3, The Loop Australia n=10), 14 of whom identified as female. The mean age of participants was 38.8 years (SD = 8.2) and all had completed at least a post-high school-level diploma, with 21 having completed a university degree.** | 6-7 |
| *Data collection* | | |  |
| 17 | Interview guide | Were questions, prompts, guides provided by the authors? Was it pilot tested?  **Semi-structured qualitative interview with prompts provided by the interviewer. The interview was not pilot tested.** | 7-8 |
| 18 | Repeat interviews | Were repeat interviews carried out? If yes, how many?  **No.** | n/a |
| 19 | Audio/visual recording | Did the research use audio or visual recording to collect the data?  **Yes.** | 7-8 |
| 20 | Field notes | Were field notes made during and/or after the interview or focus group?  **No.** | n/a |
| 21 | Duration | What was the duration of the interviews or focus group?  **Interviews ranged from 21 to 51 minutes in duration (Mean= 34 minutes; SD=9 minutes).** | 7-8 |
| 22 | Data saturation | Was data saturation discussed?  **Yes. Interviews were conducted until data saturation was obtained, as determined by consensus between the interviewers and research team.** | 7-8 |
| 23 | Transcripts returned | Were transcripts returned to participants for comment and/or correction?  **No.** | n/a |
| **Domain 3: analysis and findings** | | |  |
| *Data analysis* | | |  |
| 24 | Number of data coders | How many data coders coded the data?  **Two. The primary coder (BCD) coded all interviews and the secondary coder (NP) coded a subsample of 5 interviews (22%).** | 8-9 |
| 25 | Description of the coding tree | Did authors provide a description of the coding tree?  **No.** | n/a |
| 26 | Derivation of themes | Were themes identified in advance or derived from the data?  **Interviews were coded using the semi-structured interview protocol as a guide (deductive approach), supplemented by an inductive approach whereby the coder also allowed the creation of ideas that came-up organically, rather than in response to specific interview questions.** | 8-9 |
| 27 | Software | What software, if applicable, was used to manage the data?  **NVivo (Release 1.7).** | 8-9 |
| 28 | Participant checking | Did participants provide feedback on the findings?  **No.** | n/a |
| *Reporting* | | |  |
| 29 | Quotations presented | Were participant quotations presented to illustrate the themes / findings? Was each quotation identified? e.g. participant number  **Yes, quotations are presented to illustrate themes and findings. Quotations were identified by participant number and AOD staff role (i.e., harm reduction or treatment).** | 10-15; table 1 |
| 30 | Data and findings consistent | Was there consistency between the data presented and the findings?  **Yes. All data was examined in the context of existing research and the novel contribution of the present study.** |  |
| 31 | Clarity of major themes | Were major themes clearly presented in the findings?  **Yes.** | 10-15 |
| 32 | Clarity of minor themes | Is there a description of diverse cases or discussion of minor themes?  **While participants generally had converging opinions, there is discussion of diverging ideas among study participants, where applicable.** |  |

Interview Guide

**Co-design and acceptability testing of a drug checking brief intervention – Staff interview**

Participant ID: _______________________

Date: ______________________________

Time: ______________________________

Interviewer: _________________________

Location where the participant was recruited:

___________________________________

The following are example questions and structure for the interview portion of the study. Interview questions are designed to be used as a guide only and in a conversational manner, allowing for a discussion with the interviewee rather than a straightforward question and answer format. Open-ended questions and verbal (e.g., yeah) and non-verbal minimal encouragers (e.g., nodding) are used throughout to encourage the interviewee to express their views.

QUESTIONS

1. Tell me a little about your experience working in alcohol and other drug sector.
   - How long have you been working in this area?
   - **Where/W**hat types of **services** have you been involved with?
2. Do you have any **experience** working/ volunteering **in drug checking/ pill testing**?
   - No (go to question 4)
   - Yes
     1. **How long** have you been/were you involved in the drug checking/ pill testing space?
     2. **What drug checking initiatives** have you been involved with?
3. Take me through **what the drug checking process looks like** **in your experience**, from when the client enters the service to when they leave…
   - 1. What kind of **information** do clients receive regarding their drug sample?
        - **For example:** Presence/ absence of a nominated substance, % purity, information on harm minimisation, etc.
     2. **What do you think** about this drug checking process?
        - What works well/ doesn’t work well for **staff**?
        - What works well/ doesn’t work well for **clients**?
        - Is there any aspect of the process/ feedback that clients or staff find particularly **helpful/ unhelpful**?
        - Is there **anything missing** from this drug checking process in your opinion?
     3. Are there any **changes** that should be made to the process to **suit different groups** of clients?
        - People seeking treatment for their substance use
        - People who inject drugs
        - People who primarily use drugs in the context of parties/festivals/pubs and clubs
        - People who primarily use performance and image enhancing drugs (e.g., steroids, selective androgen receptor modulators (SARMS)).
     4. In your experience, **how long do clients want to spend** **talking** to workers about their substance use while going through the drug checking process?

*If the participant does not have experience with drug checking, read the below scenario:*

**Drug checking is the process of testing a drug (any drug, not just pills) voluntarily submitted by people who use drugs (not the police or government), to determine what substance(s) it does/ does not contain, and depending on the technology, to determine drug purity and whether the drug has been cut/ mixed/ adulterated with anything else. Results of the tests are provided back to a service user by a health professional. Trials of drug checking services at festivals have recently been conducted in Australia (i.e., at the 2018 and 2019 Groovin the Moo festivals), and the ACT recently opened a fixed-site drug checking service (i.e., CanTEST Health).**

1. **How likely** do you think it is that people would use a drug-checking service if it were available? Why/ why not?
   - People seeking treatment for their substance use
   - People who inject drugs
   - People who primarily use drugs in the context of parties/festivals/pubs and clubs
   - People who primarily use performance and image enhancing drugs (e.g., steroids, selective androgen receptor modulators (SARMS)).
2. In your opinion, what would be some of the **barriers** to accessing a drug checking service if one were available?
3. In your opinion, what would **make it easier** or make people more likely to access a drug checking service?
4. **What kind of information** do you think clients accessing a drug checking service should receive about the substance being submitted for checking?
   - **For example(s):** Information regarding how many people their age and sex use the drug they’re testing, Information regarding the health risks associated with the drug they’re testing, harm minimisation tips, etc.
5. **We will now read you a hypothetical scenario which describes the process a client would undergo when accessing a drug checking service…**

**The client is asked to place a small amount of powder or part of a tablet / capsule (about 10mg/ one tenth of a point) into a clip seal bag, which is dropped into a locked box.**

**
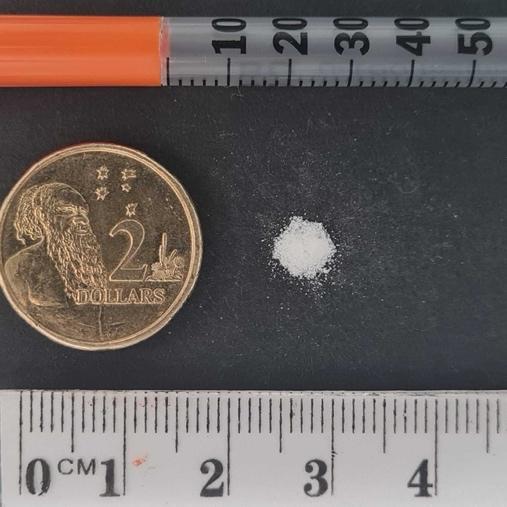
**

**The client is asked to complete a short anonymous questionnaire asking the following questions:
1) What they think the substance is**

**2) Have they already tried some of this substance, and if so where the effects as expected / not as expected**

**3) Are they planning to inject this substance?**

**The chemist will then analyse the substance which takes about 15-20 minutes. A health professional then conducts a brief intervention which collects non-identifiable information about the client including their age, gender, cultural background, and employment status, and includes an alcohol and other drug use assessment, and a general health assessment (which includes…). The health professional then provides the client with the results of the chemical analysis and information on harm reduction strategies relevant to the client’s individual needs. This takes about 15-20 minutes.**

- - 1. **What do you think** of this drug checking process?
       - Any aspects you **like/ don’t like** about the process?
       - Anything you think would be particularly **helpful/ unhelpful**?
       - Anything you would **change about/ add to** the process?
       - Anything **missing** from the process?
    2. Are there any **changes** that should be made to the process to **suit different groups** of clients:
       - People seeking treatment for their substance use
       - People who inject drugs
       - People who primarily use drugs in the context of parties/festivals/pubs and clubs
       - People who primarily use performance and image enhancing drugs (e.g., steroids, selective androgen receptor modulators (SARMS)).

1. The next few questions ask about your thoughts on colour reagent tests which are sometimes used in drug checking services. [if clarification is needed: Colour reagent drug checking works by mixing a small scraping from a drug (<.01 grams/ one tenth of a point) with a specific liquid (determined based on the presumed class of the drug being tested). The liquid, which changes in colour after mixing with the drug, is then compared to a reference chart to determine the presence/ absence of the presumed substance.]
   - Do you have **professional experience** with colour reagent tests for drug checking?
   - In your opinion, what are the **practical strengths and limitations of colour reagent tests** for drug checking? (e.g., time, cost, client preference, etc.)
   - **What do you think** of the use of colour-reagent tests in drug checking services?
2. The next few questions ask about your thoughts on other drug checking methods such (e.g., spectrometry), which can provide more detailed information than colour-reagent tests, including information on drug purity, and the number and quantity (i.e., percentage) of drugs and adulterants (i.e., other substances cut/mixed with the drug) present in a drug sample. While these tests can generally provide more detailed information, they are much more expensive than colour reagent tests and take a longer time to process as this method requires specialised equipment and a person with expertise in the testing method to analyse the drug sample. So, the wait time for results using this method can range from between 1 hour up to 3 weeks, depending on how common the drugs being tested and the mixtures within the sample are.
   - In your opinion, what are the **practical strengths and limitations of more sensitive methods** for drug checking? (e.g., time, cost, client preference)
   - **What do you think** of the use of more sensitive methods in drug checking services?
3. Do you have **any** **concerns** about drug checking being offered in your service?
4. Is there **anything else that we didn’t cover** during the interview regarding drug checking that you would like to comment on/ that you think we should consider?

**The Research Team**

All authors have experience in substance use research, while BCD and LH have clinical experience working with people with substance use disorders, and CP and CF have experience in drug checking. NP, BCD, CP, DS, and LH are employed as university-based researchers, with NP, BCD, and LH working within a team that has established research collaborations with LLW. CP and CF are members of The Loop Australia. Importantly however, the interviewers and those involved in analysing the interview data did not have established relationships with any of the study participants. Nonetheless, the combined authors’ experiences in substance use research, treatment, and drug checking likely shaped the study from the creation of the qualitative interview to the analysis of that data. Finally, it is important to also acknowledge that all authors are university-educated (e.g., Masters or PhD in psychology, public health, or health studies) and their experience with substance use research, treatment, and drug checking stem from a Western cultural context which also likely influenced interpretation of the qualitative data.

Table S2. Themes and example quotes from the qualitative interviews

| **Theme 1**: **People who use drugs (PWUD) infrequently are more likely to access a drug checking service than people who are dependent on drugs** |
| --- |
| *So, a lot of clients are like I don’t, ‘I don’t OD because I've been doing this for 25 years, every single day’… So the perceived risk is not there.”* – 48 years, AOD harm reduction staff  *… There would be certain cohorts that would use [drug checking] more readily than others in the substance user community. The predominant cohort, I would expect are the ones that are more into the festival or the party scene. And often that's because they're not having a regular supplier as such, because it's often in a more intermittent periodical use pattern, which means that they may be trying to contact a couple of suppliers and therefore that long standing relationship with a supplier that often is the case with someone who has a regular substance using profile won't be there, which means that there's less trust to say that the quality of the substances is going to be okay.* – 39 years, AOD harm reduction staff  *Sometimes just giving up your stuff, no matter how beneficial it’s going to be, can be really, really challenging, especially if someone's at the higher end of a substance use habit … And they're often balancing quite a high level of risk, as well in terms of overdose or withdrawal being a part of it if they're not hitting that right amount. And the cost that it requires to get to that amount has gone up quite a bit. So the idea of giving away anything, when it's so valuable to their well-being can be an internal barrier.* – 39 years, AOD harm reduction staff  *Yeah, I think I think just witnessing people who are basically coming into the [safe injecting service], and they they’re feeling really sick, so that they’re hanging out, and they’re withdrawing. They want to get into the service really, really quickly. And I think if there’s an additional step to come in and be like, I’m gonna test my drugs before I use that, that’s too much time because I might have to wait to actually come into the room anyway. I think that it’s just the addictive drive and the needing to not feel the withdrawals, it’s more important than knowing what’s in [their drugs]. That would, yeah, I think there might be some people [that might use drug checking] but if you’re feeling really, really sick, yeah, I don’t think I don’t think it would be top priority.* – 41 years, AOD harm reduction staff  *And so people, again, the people who tend to come and use in the [safe injecting] room in that way seem to be ... there’s a desperation. There’s a you know, dependence. Like it’s, it’s an there’s an intensity about the use, and I feel like that’s ... checking drugs wouldn’t be a priority for them.* – 48 years, AOD harm reduction staff |
| **Theme 2: Confidentiality and anonymity concerns are the greatest barrier to individuals accessing a drug checking service** |
| *I think, obviously, [a barrier to accessing drug checking would be] feeling like they’d be potentially monitored by the police, or that there’s some sort of record around who’s using and who’s not using, who’s coming in and using the service. So, I think confidentiality would be a barrier and like the fear of legal consequences.* – 36 years, AOD harm reduction staff  *I think the police factor is ... unless there’s a clear line and a clear communication and culture from police ... that are supportive in that space, many services users, that’s going to be a barrier for them, because ... a lot of them will have had bad experiences with police.* – 34 years, AOD treatment staff  *... [Something that would facilitate use of a drug checking service is if it] was in a discreet and confidential location, where clients wouldn’t feel that it was, you know, they could be seen by other members of the public ...* – 41 years, AOD treatment staff  *I think [people accessing drug checking] depends… how much confidence they had in the robustness of the confidentiality, you know, so as long as they were not asked for a name, or that they could do it completely anonymously, and privately, and no where near police or even security, probably.* – 50 years, AOD treatment staff  *You know, like in prison where you've got cubicles to visit people. You could go and go into a cubicle and shut the door, and nobody would know you’re in there, basically … You come in one way and you go out the other way. So then you’re not crossing paths [with other clients].* – 41 years, AOD harm reduction staff |
| **Theme 3: Ease of use is integral to drug checking uptake** |
| *… I think festivals or nightclub precincts, having a little service there would be good, really good.”* – 42 years, AOD treatment staff  *…Co-location for [people who are dependent on drugs] is going to be your best option with needle exchange programs. I think that they have kind of already that familiar setting. It’s a semi-trusted service for most clients in active use as well.”* – 30 years, AOD treatment staff  *Put [drug checking services] at the festivals. … In a nightclub area… I think you’re gonna get more people coming to use the services and have their drugs tested, because this is the cohort that are going to use it.* – 52 years, AOD harm reduction staff  *And I do believe that if the tests like the time between testing and the time between having that conversation, honestly, if it wasn't within the in 30 minutes, I don’t know if they’ve got the patience, either.* – 26 years, AOD treatment staff  *That kind of that is one of my concerns, you know, that we would have substances on-site [at a drug and alcohol treatment service]. And while there are people who are trying to be abstinent, so, you know, how that works in reality? I don’t know.* – 50 years, AOD treatment staff |
| **Theme 4: Creating a safe, non-judgemental environment is critical to the uptake of drug checking services and can be fostered through the inclusion of peer workers with lived experience** |
| *I think a peer worker and AOD nurse would be really good together. Just to have that mix, so maybe the peer worker doing the initial explaining the process … And then referring [the client] to the nurse or to medical resource …* – 38 years, AOD harm reduction staff  *Well I think the fear factor is a big [barrier]. Like, I think just having peers in an organisation to sort of direct it – to keep it sort of dynamically linked in with service users needs can be a really good thing in an organisation …*  *There’s I suppose like a balance between professionalism and like a well-boundaried professionally held space, but that also doesn’t have that sort of like 'us and them', power divide type thing … I guess it just really comes down to the attitudes of the people working in the space. And whether they’re able to create a, you know, a place that feels safe and non-judgmental, and supportive, and all those sorts of things. I mean, it’s a, it’s a night and day difference when people are able to sort of do that and hold that space for people to use drugs as opposed to not and I mean, we hear so often of people having negative experiences with GPs and psychologists and psychiatrists and all that kind of stuff, you know?* – 36 years, AOD harm reduction staff |
| **Theme 5: People who sell drugs will likely use a drug checking service, with mixed perceptions of the consequences** |
| *[Drug dealers] will they tell their customers “Hey, this one's stronger. Be careful you know, I’ve had such and such OD”. They’re usually pretty good about – pretty passionate about the quality of their stuff and they’ve got the money to pay for the test so … that information would very quickly go out to the people that are buying it ... Yeah, because people don’t want someone Oding ... Yeah, I just think that would filter down to everyone ... struggling addicts that are doing it really tough probably wouldn’t be able to afford [drug checking], but, but the benefits would come down to them.* – 38 years, AOD harm reduction staff  *The only thing I have wondered, I suppose is whether a fixed site service, not on-site at a festival, would potentially have dealers as their clientele. Just checking that their stuff is okay. Just I’d be super curious as to whether that would occur and if there would be any sort of benefit to that … I know that there is evidence to suggest that drug checking has impacted black markets a little bit. So, I actually think there might end up being a little bit of that going on, which would be really cool* – 33 years, AOD harm reduction staff  *Yeah, so one of the areas that I work quite a lot in is the inclusion of people who sell drugs and drug checking services. And by that, I mean, like, you know, working with people who sell drugs to kind of get a handle or maybe surveillance on the unregulated market. So I think it’s a very tricky area, because often people who sell drugs fall outside of drug policy stuff and that's always seen as illegal and damaging ... Often they can often offer like valuable insight into drug markets.* – 32 years, AOD harm reduction staff  *Could the service be like misused in the sense of like a dealer who’s got a lot from the same quote unquote cook or batch who would want to say check that what they’re about to wheel and deal is what they say it is and be able to almost use that as drumming up business … I think I was thinking of Breaking Bad [television series]. I wondered if there was a potential then for [drug checking] to be kind of misused.* – 26 years, AOD treatment staff |
